# Lessons From CABBI: Advancing Vegetative Oil Production in C_4_ Crops Through a Vertically Integrated DBTL Framework

**DOI:** 10.1111/pbi.70771

**Published:** 2026-10-28

**Authors:** Daniel Lunn, Kiyoul Park, Narendra Naik Deshavath, Jia Dong, Kankshita Swaminathan, Jeremy Guest, Andrew D. B. Leakey, Nathaniel M. Butler, Jorg Schwender, Vijay Singh, John Shanklin, Fredy Altpeter, Edgar B. Cahoon

**Affiliations:** ^1^ Carl R. Woese Institute of Genomic Biology University of Illinois Urbana‐Champaign Urbana Illinois USA; ^2^ Department of Plant Biology University of Illinois Urbana‐Champaign Urbana Illinois USA; ^3^ Center for Digital Agriculture University of Illinois Urbana‐Champaign Urbana Illinois USA; ^4^ DOE Center for Advanced Bioenergy and Bioproducts Innovation University of Illinois Urbana‐Champaign Urbana Illinois USA; ^5^ Center for Plant Science Innovation and Department of Biochemistry University of Nebraska‐Lincoln Lincoln Nebraska USA; ^6^ DOE Center for Advanced Bioenergy and Bioproducts Innovation University of Nebraska‐Lincoln Lincoln Nebraska USA; ^7^ Department of Agricultural and Biological Engineering University of Illinois Urbana‐Champaign Urbana Illinois USA; ^8^ Plant Breeding and Genetics Section, School of Integrative Plant Science Cornell University Ithaca New York USA; ^9^ HudsonAlpha Institute for Biotechnology Huntsville Alabama USA; ^10^ DOE Center for Advanced Bioenergy and Bioproducts Innovation HudsonAlpha Institute for Biotechnology Huntsville Alabama USA; ^11^ The Grainger College of Engineering, Department of Civil and Environmental Engineering University of Illinois Urbana‐Champaign Urbana Illinois USA; ^12^ Department of Agronomy & Horticulture University of Nebraska‐Lincoln Lincoln Nebraska USA; ^13^ Plant Transformation Core Research Facility, Agricultural Research Division, Institute of Agriculture and Natural Resources University of Nebraska‐Lincoln Lincoln Nebraska USA; ^14^ Department of Biology Brookhaven National Laboratory Upton New York USA; ^15^ DOE Center for Advanced Bioenergy and Bioproducts Innovation Brookhaven National Laboratory Upton New York USA; ^16^ Agronomy Department, Plant Molecular and Cellular Biology Program, Genetics Institute University of Florida, Institute of Food and Agricultural Sciences Gainesville Florida USA; ^17^ DOE Center for Advanced Bioenergy and Bioproducts Innovation University of Florida Gainesville Florida USA

## Abstract

The global demand for renewable liquid fuels and biobased chemicals has intensified interest in engineering high‐biomass C_4_ grasses to accumulate triacylglycerol (TAG) in vegetative tissues. Achieving this goal requires coordinated solutions across molecular biology, crop science, chemical engineering, and life cycle assessment, disciplines that rarely operate within a single research program. The Center for Advanced Bioenergy and Bioproducts Innovation (CABBI) was established to provide this integration through a Design–Build–Test–Learn (DBTL) framework applied to three C_4_ feedstocks: saccharum (*Saccharum* spp. hybrids), sorghum ( *Sorghum bicolor* ), and miscanthus (*Miscanthus* × *giganteus*). Through iterative DBTL cycles, CABBI has produced transgenic saccharum and sorghum lines with substantially elevated levels of vegetative TAG, with multi‐location field trials having confirmed stable trait heritability and lipid yields. We have also leveraged our integration to develop a dual system biorefinery that co‐recovers vegetative lipids with fermentable sugars at levels sufficient to achieve industrial ethanol titres. Importantly, this fully integrated cross‐disciplinary data integration model has highlighted the key constraints currently preventing TAG‐rich vegetative oil from achieving commercial viability as: (i) active lipid turnover, (ii) photosynthetic trade‐offs, (iii) spatial misexpression, and (iv) bioprocessing challenges. CABBI uses emerging technologies, including biofoundry‐enabled construct screening, precision genome editing, and machine‐learning‐assisted regulatory design, to further accelerate DBTL cycles. More broadly, this work illustrates how integrative centre‐scale research that unites diverse expertise can accelerate discovery and translation, positioning these systems to expand agricultural markets and contribute to a more secure and resilient bioeconomy.

## 1 Introduction: The Imperative for Integrated Innovation

The Center for Advanced Bioenergy and Bioproducts Innovation (CABBI) is a United States Department of Energy (DOE) Bioenergy Research Center. One key CABBI objective is engineering crops to produce oil in vegetative tissues. This vegetative oil, primarily in the form of triacylglycerol (TAG), is a major feedstock target for CABBI, since it contains a plethora of common and unique fatty acid species that impart chemical properties useful for platform chemicals, biomaterials, bioproducts, and biofuels (Metzger et al. 2025). In particular, the demand for biofuel production in the US has significantly increased in recent years due to state and federal policies and tax incentives. The US Environmental Protection Agency, for example, updated the Renewable Fuel Standard in 2026 to mandate further increases in the minimum levels of biofuel blending in transportation (U.S. EPA 2026). Further, the Section 45Z Clean Fuel Production Credit and the Advanced Energy Project Credit provide financial assistance for biofuel production, infrastructure development, and reequipping existing refineries (Bistline et al. 2023). A major target for biofuels is to increase domestic energy supply, with a focus on liquid fuels for maritime shipping, aviation, and heavy‐duty land transport, including commercial trucking and agricultural machinery. This need arises due to a fundamental energy density gap in lithium‐ion batteries. While electrification is sufficient for light transport, lithium‐ion batteries have an energy density ~2% that of diesel, making them less suited for long‐haul transportation (Gray et al. 2021). However, liquid biofuels have an energy density of around 90% of petrochemical fuels, making them an attractive option particularly for shipping and aviation companies seeking to lower emissions (He et al. 2025; Holladay et al. 2020).

Oilseed plants have traditionally served as the primary source of TAG for biofuel production. However, seed oil yield is inherently limited due to the partitioning of carbon into specialized reproductive structures (Gautam et al. 2025). A solution to this limitation is to redirect carbon flux toward TAG synthesis in vegetative tissues. Initial modelling suggested that if crops can accumulate TAG at 10% of their total above‐ground dry biomass, this would provide an order‐of‐magnitude higher yield than currently achieved in oilseeds (Durrett et al. 2008; Park et al. 2021). These yields could be achieved and further increased by producing vegetative TAG in high‐biomass C_4_ grasses. These species can achieve exceptional dry matter yields of 20–70 Mg ha^−1^ per year, with estimates of oil production reaching 3 t ha^−1^ per year (Byrt et al. 2011; Heaton et al. 2008; Matsuoka et al. 2014; Rooney et al. 2007). Additionally, this approach would enable a ‘dual stream’ biorefinery model that extracts TAG for liquid biofuels and platform chemicals while simultaneously saccharifying and fermenting the residual lignocellulosic biomass to ethanol and other target products. Lastly, techno‐economic analysis supported the potential of this approach, with data suggesting that 2% TAG accumulation in sugarcane vegetative tissue would achieve oil production levels comparable to those of soybean (Huang et al. 2016; Maitra et al. 2024).

CABBI aims to overcome a key obstacle to developing these technologies in academia by providing a vertically integrated infrastructure that individual laboratories cannot provide. This integration enables CABBI to implement a ‘lab‐to‐field‐to‐bioprocessing’ pipeline that addresses the biological and engineering challenges of producing and processing vegetative TAG across multiple scales. The target feedstock goal is to engineer oil production in the vegetative tissues of high‐biomass C_4_ grasses for renewable diesel, sustainable aviation fuel, platform chemicals, and bioproducts. Engineering these crops to produce vegetative TAG requires integrating molecular biology, metabolic modelling, genomics, transformation technologies, physiology, and agronomy to develop an integrated pipeline that feeds pilot‐scale bioprocessing facilities. This vertical integration allows the production of TAG‐accumulating feedstock crops to be assessed across the entire life cycle, from metabolic engineering to multisite field evaluation in real‐world conditions to biomass deconstruction.

CABBI implements a Design‐Build‐Test‐Learn (DBTL) framework to translate metabolic insights into improved vegetative oil crops and processes (Figure 1). CABBI integrates crop science, molecular biology, chemical engineering, and life cycle assessment into a research system to implement iterative DBTL cycles. The importance of this DBTL framework lies in revealing the inherent complexity of producing vegetative TAG in C_4_ grasses. Many of the current constraints to achieving high vegetative TAG production and processing were identified only through these multi‐scale DBTL cycles. Here, we present CABBI's progress toward producing vegetative oil and discuss how lessons learned from these cycles offer broader perspectives regarding the value of vertically integrated research for advancing complex traits in crop species.

**FIGURE 1:**
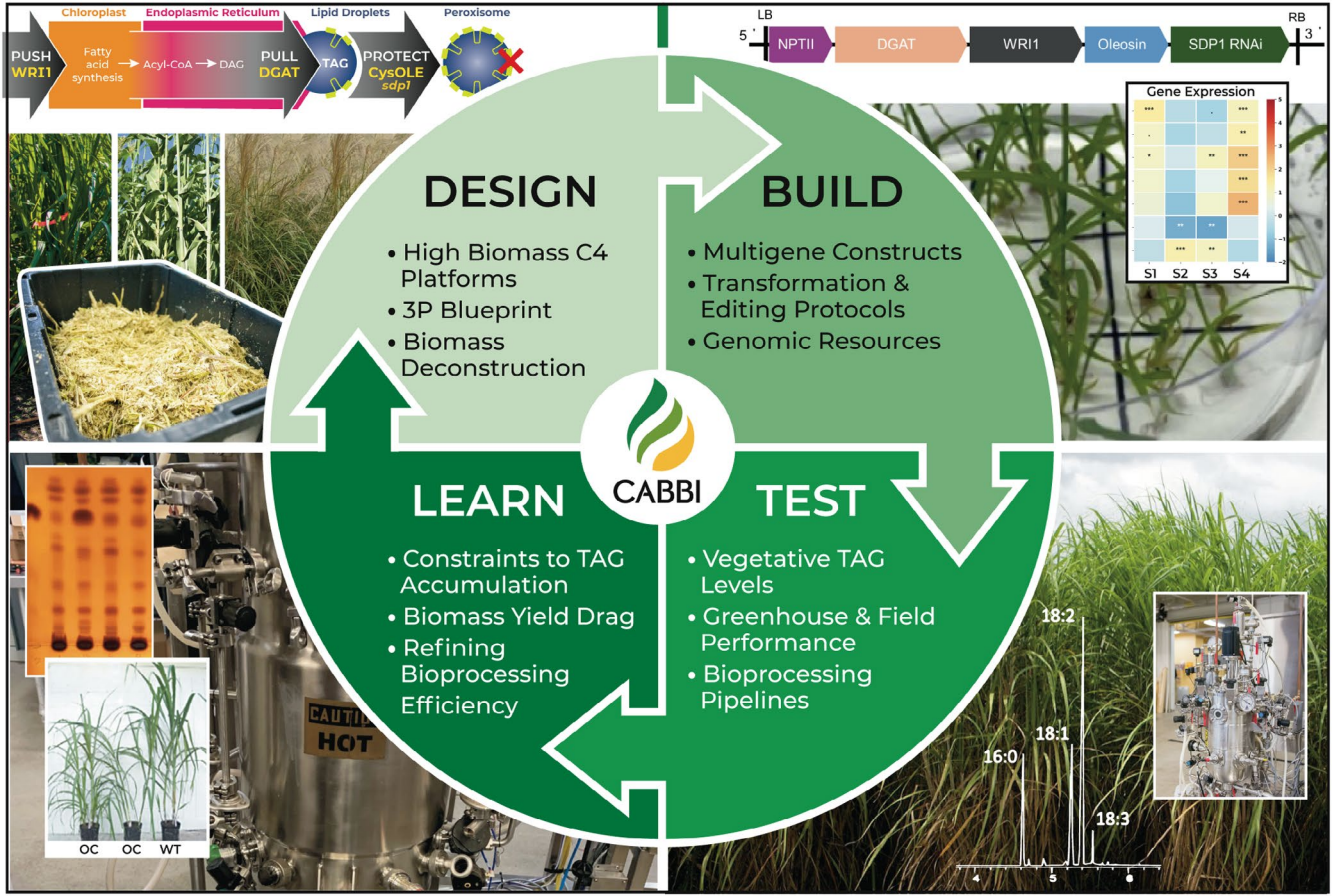
Design‐build‐test‐learn cycle. A graphic representation of the iterative design‐build‐test‐learn cycles at CABBI. Arrows show the directionality associated with this engineering strategy. Text highlights key progress made within each step of the ‘DESIGN’, ‘BUILD’, ‘TEST’, ‘LEARN’ framework at CABBI.

## 2 DESIGN: Strategic Architecture for a Mission‐Oriented Bioeconomy

The ‘DESIGN’ phase at CABBI occurs in tandem at multiple scales to ensure that feedstocks and metabolic engineering designs remain compatible with biomass deconstruction, large‐scale recovery, and conversion. This strategy enables the optimization of feedstock biology and bioprocess engineering, as the demands of economically viable industrial deconstruction determine the biological traits targeted in feedstock design. CABBI addresses this by integrating genomic technologies, molecular biology, and chemical engineering, providing a model for developing sustainable biofuels without competing with existing agricultural systems.

### 2.1 Feedstock Design: Selecting High‐Biomass C_4_ Platforms

The first critical design decision is selecting crops to avoid competition between food and fuel production systems. Avoiding this conflict is critical because the USDA already estimated that more than half of US soybean production was diverted to biodiesel in 2025 (Debnath et al. 2025), which equates to approximately 45 million acres of prime agricultural land. Recognizing this, CABBI focuses on improving three complementary high‐biomass C_4_ crops, which are all suited to take advantage of the marginal lands available in the rainfed USA (He et al. 2024). Our monocot grass targets are *Miscanthus* × *giganteus* (miscanthus), *Sorghum bicolor* (sorghum), and select *Saccharum officinarum* hybrids (saccharum). These crops achieve high biomass levels through the C_4_ photosynthetic pathway and exhibit exceptional water and nitrogen use efficiencies (Byrt et al. 2011; Zhu et al. 2008). These features allow our feedstock portfolio to achieve 20–70 Mg dry weight ha^−1^ yr^−1^ (Heaton et al. 2008; Mathur et al. 2017; Matsuoka et al. 2014) without competing with food production systems.

Geographically, these feedstocks can be grown across a broad range of environments throughout the United States. Energycane is an interspecific hybrid of *S. spontaneum* L. and *S. officinarum* that thrives in the Southeastern US and tropical zones; the sterile triploid hybrid *Miscanthus × giganteus* is ideally suited for temperate regions in the US Midwest and Northeast, while drought‐tolerant sorghum complements the portfolio due to its broad adaptation to semi‐arid areas across the US. Adding sorghum to the portfolio also offers economic benefits by providing biorefineries with an alternative feedstock when oil‐producing sugarcane is out of season or if perennial stands are unexpectedly destroyed (Cortés‐Peña et al. 2022). Beyond biomass, these species provide ecosystem benefits, including soil carbon sequestration, reduced erosion, and decreased nutrient leaching through their extensive root systems (Blakely et al. 2025; He et al. 2024; Moore et al. 2021). These ecosystem features are also economically advantageous, as they benefit from federal and state incentives and tax credits that support the financial viability of second‐generation biorefineries (Stewart et al. 2023, 2025). Collectively, these characteristics make sorghum, energycane, and miscanthus ideal production platforms for channelling photosynthetically fixed carbon into energy‐dense TAG.

### 2.2 Metabolic Design: Implementing the 3P Blueprint

Designing crops for vegetative oil accumulation requires a significant departure from traditional seed‐based metabolism. Increasing seed TAG levels or engineering them to have a value‐added fatty acid composition has long been a goal in oilseed research (Cai et al. 2022; Lunn et al. 2022). Unfortunately, this approach has proved difficult in seeds, requiring large multigene constructs to increase TAG accumulation and successfully shuttle non‐natively produced fatty acids through lipid assembly pathways (Lunn et al. 2019, 2020). Furthermore, the presence of value‐added fatty acids in seed TAG has often led to developmental defects during plant establishment, as native plant machinery struggles to use non‐native TAG species (Lunn et al. 2018). Consequently, CABBI has prioritized the production of TAG in vegetative tissues to achieve high levels of accumulation and avoid unwanted detrimental developmental defects.

At CABBI, we create expression cassettes using modular cloning technologies that express transgenes or modulate target gene expression. Introduction of these construct designs into the target species then occurs via biolistic or Agrobacterium‐mediated strategies. TAG accumulation in oilseed metabolism occurs through three distinct metabolic pathways: fatty acid biosynthesis, triacylglycerol assembly, and lipid droplet accumulation (Cao, Kannan, et al. 2023). Early research into engineering oil accumulation in vegetative tissues provided proof of principle for a framework called ‘Push, Pull, and Protect’ (3P) (Vanhercke et al. 2014).

CABBI has built upon this by deploying constructs that use a streamlined 3P strategy optimized for the specific challenges of C_4_ grass tissues (Kannan et al. 2022; Parajuli et al. 2020; Park et al. 2021) (Figure 2). In this blueprint, the ‘Push’ factor uses the transcription factor WRINKLED1 (WRI1) to activate genes in central metabolism and fatty acid synthesis (Cernac and Benning 2004). The ‘Pull’ component employs DIACYLGLYCEROL ACYLTRANSFERASE (DGAT) to catalyse the final and rate‐limiting step in TAG assembly (Zou et al. 1999). ‘Protect’ occurs by manipulating either individual or a combination of OLEOSIN1 (OLE1), which coats lipid droplets to sterically hinder lipase access (Siloto et al. 2006), suppression of sugar‐dependent 1 (SDP1), which prevents the first committed step in TAG breakdown (Eastmond 2006), or suppression of TRIGALACTOSYLDIACYLGLYCEROL1 (TGD1), which prevents lipid transfer from the endoplasmic reticulum to the chloroplast, leading to a retension of diacylglycerol in the ER, where it is available as substrate for TAG synthesis.

**FIGURE 2:**
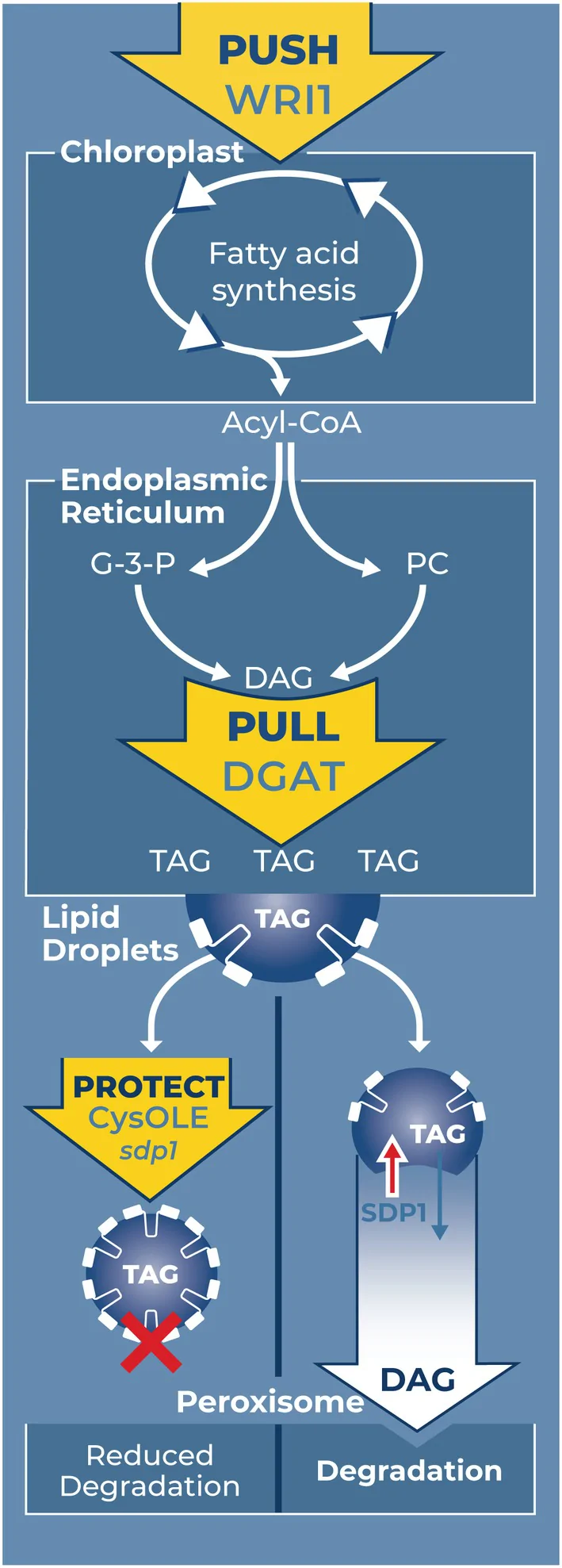
The 3P metabolic blueprint. Graphic representation of the 3P blueprint design to produce vegetative TAG in CABBI feedstock crops. The graphic shows the location of the ‘Push, Pull and Protect’ within the plant cell. Genes associated with each of the 3Ps are highlighted in yellow. Key lipid intermediates in TAG biosynthesis are G‐3‐P (glycerol‐3‐phosphate), PC (phosphatidylcholine), DAG (diacylglycerol). Red crosses depict how the protect step prevents lipid droplets containing TAG from entering the peroxisome.

### 2.3 Bioprocess Design: Planning for Biomass Deconstruction

The final pillar of the design phase is to maximize product yield per ton of biomass through bioprocessing. CABBI develops oil‐accumulating feedstocks in parallel with industrial processes that release fermentable sugar while protecting lipid integrity (Figure 3). Therefore, selecting the appropriate pretreatment to deconstruct the lignocellulosic matrix is critical to limit sugar degradation and the formation of inhibitors such as furfural, 5‐HMF, formic acid, and levulinic acid (Deshavath, Woodruff, Eller, et al. 2024). With these considerations in mind, CABBI has developed BioSTEAM software, which enables biorefinery design, simulation, and techno‐economic analysis to inform design decisions and scenarios (Cortes‐Peña et al. 2020).

**FIGURE 3:**
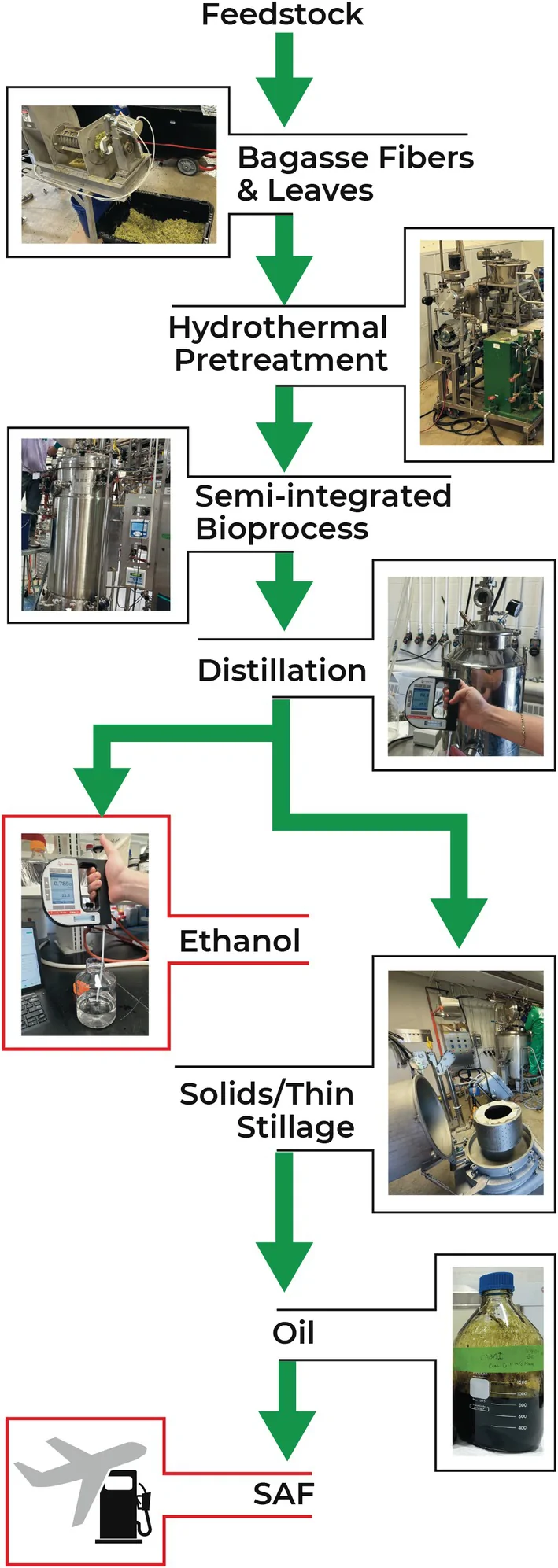
Designed pipeline for bioprocessing. Graphic representation of the pipeline for bioprocessing depicting each major stage with oil, sustainable aviation fuel (SAF), and ethanol production as end products from bagasse and leaves of CABBI feedstocks.

CABBI research conducted with other Department of Energy Bioenergy Research Centers demonstrated that hydrothermal pretreatment is the optimal chemical‐free approach for preserving oil and its recovery from CABBI transgenic crops, whereas saponification of lipids occurred with alkaline and ionic‐liquid pretreatments, greatly affecting the lipid recovery (Deshavath et al. 2025). This pretreatment hydrolyzes ester bonds in neutral lipids without affecting the enzymatic degradation of intracellular lipids and thereby enriching the total fatty acid content (Maitra et al. 2024). Further, our experiments have found that bioprocessing may not require tailoring extraction strategies to the specific morphology of the feedstock. CABBI found that removing leaves before juice extraction is the preferred design despite saccharum and sorghum species having different leaf arrangements (Deshavath, Woodruff, and Singh 2024; Maitra et al. 2024). This treatment leads to greater yield by preventing sorption by leaves and diluting sugar concentrations through mass‐transfer gradients. Collectively, hydrothermal pretreatment paired with pre‐removed leaves forms a robust preprocessing workflow for extracting high‐value lipids and sugars from CABBI feedstocks.

## 3 BUILD: Developing Genetic Architecture for High Vegetative TAG‐Producing Biomass Crops

The ‘BUILD’ phase at CABBI aims to develop vegetative TAG‐accumulating crops that outperform those from conventional breeding. This requirement necessitates parallel efforts to refine genomic resources, transformation technologies, and exogenous DNA delivery across three distinct feedstock platforms. The inherent complexity of sugarcane, sorghum, and miscanthus has historically hindered rapid genetic improvement. All three species are also highly recalcitrant to tissue culture, making it difficult to regenerate whole plants from engineered cells. Lastly, significant challenges still remain in delivering large, multigene constructs to coordinate the 3P blueprint in these crops. Addressing these collective challenges is vital for creating a transformation infrastructure to accelerate DBTL cycles and has been a major focus in early experiments.

### 3.1 Sorghum: The Proof‐Of‐Concept Annual Diploid

As an annual diploid crop with robust genetic and genomic resources, sorghum serves as a critical tool for proof‐of‐concept work within CABBI. Sorghum also enables accelerated DBTL cycles and lab‐to‐field experiments through seed‐based transport of transgenics and government‐regulated field trials, compared to saccharum and miscanthus. Sorghum enables CABBI to use Agrobacterium‐mediated construct delivery to facilitate high‐throughput vector assembly, enabling the recovery of low‐copy, intact gene cassettes spanning 18–20 kb (Nguyen et al. 2007). While immature embryo transformation remains the gold standard for specific grain and sweet varieties, this process still takes between 8 and 12 months (Butler et al. 2025). Therefore, accelerating the efficiency and time for sorghum transformation is a major goal of CABBI to increase the frequency of DBTL cycles. A previous breakthrough in sorghum transformation was the ability to utilize leaf material instead of immature embryos (Wang et al. 2023). CABBI built upon this success by adopting monocot RNA viruses, such as Foxtail Mosaic Virus (FoMV), to deliver morphogenic regulators that resulted in between 75% and 79% formation of embryonic calli (Butler et al. 2025). Combined with the leaf transformation method, these morphogenic regulators significantly expand the range of sorghum varieties available for transformation. This transformation pipeline was then successfully deployed, showing greatly improved delivery of gene‐editing reagents and reduced transformation time by around 3 months (Baysal et al. 2025). CABBI also generated the first transgene‐free CRISPR‐edited sorghum, which produced altered leaf inclination through mutagenesis of *LIGULELESS‐1* to increase light interception in dense planting (Brant et al. 2021). These advances expand existing genome‐engineering resources and establish sorghum as a relatively short and easily transformed crop for testing construct designs to improve TAG accumulation.

### 3.2 Saccharum: Engineering Complex Polyploids

Constructing oil‐accumulating saccharum hybrids, like energycane, required overcoming extreme recalcitrance to transformation. Significant progress in this area was made by identifying suitable genotypes, refining the chosen culture media and improving gene delivery (Luo et al. 2022; Cao, Luo, et al. 2023; Brant et al. 2025). When exploring delivery methods, side‐by‐side comparisons of biolistic particle bombardment and *Agrobacterium*‐mediated gene transfer both offer similar transgene integration complexity, expression levels and stability (Jackson et al. 2013; Wu et al. 2015). However, biolistic gene transfer is often preferred for sugarcane and energycane because it facilitates the co‐delivery of multiple unlinked gene constructs required for complex metabolic engineering and offers superior field performance compared to *Agrobacterium*‐mediated transformation due to a shorter tissue culture period (Cao, Kannan, et al. 2023; Joyce et al. 2014).

Establishing sophisticated CRISPR/Cas9 technologies for sugarcane underscores the strength of the vertically integrated research model at CABBI (Altpeter 2025). CABBI has reported the first successful targeted mutagenesis of the complex sugarcane genome with CRISPR/Cas technology (Eid et al. 2021), followed by the first report of precise gene editing that achieved multi‐allele nucleotide substitutions (Oz et al. 2021). Further, we have developed CRISPR interference (CRISPRi) technologies that achieve up to 82% gene repression, thereby enabling modulation of gene activity rather than complete elimination (Hooghvorst and Altpeter 2023). These advances led to the first field trial of CRISPR‐edited sugarcane, in which the scalable co‐editing of *LIGULELESS‐*1 in this highly polyploid crop allowed the fine‐tuning of leaf inclination angle, enabling the selection of an ideotype to potentially improve biomass yield by 18% dry weight (Brant et al. 2021, 2024). Developing these technologies represents a major milestone and significantly enhances the toolkit for sugarcane gene editing while providing CABBI a superior chassis to increase TAG accumulation.

### 3.3 Miscanthus: Breaking the Transformation Barrier

CABBI played a significant role in overseeing the development of transformation and gene editing procedures for miscanthus. Prior to CABBI efforts, biotechnological improvements in miscanthus were limited to random, untargeted transgene insertions. This is because miscanthus species are paleo‐allopolyploids carrying duplicated regions of ancient, sorghum‐like DNA, resulting in highly syntenic homoeologous chromosomes that lead to extreme functional redundancy (Swaminathan et al. 2012). Therefore, achieving a true ‘knockout’ phenotype requires guide RNAs designed to target all homeologs and their respective alleles simultaneously. CABBI successfully sequenced the miscanthus genome (Mitros et al. 2020). Leveraging this, CABBI established and optimized a transformation pipeline for genome editing of miscanthus that showed biolistic transfer produced higher delivery efficiencies than *Agrobacterium* (Trieu et al. 2022). Here, CABBI researchers used a multi‐step screening pipeline to design gRNAs that targeted the highly conserved *LEMON WHITE 1* (*LW1*) gene (Lu et al. 2012), which is essential for chlorophyll and carotenoid biosynthesis to demonstrate genome editing. By transforming embryogenic calli clusters of undifferentiated cells with the potential to become embryos, CABBI successfully produced edited plants across three miscanthus species and five different genotypes. Visual confirmation of these edits was provided by pale green, yellow, or solid white stripe leaf phenotypes as expected of the lw1 gene knockout (Trieu et al. 2022). This study demonstrates the feasibility of genome editing and lays the foundation for designing miscanthus that accumulate vegetative TAG.

## 4 TEST: Validating Transgenic Designs for Oil Production and System Performance

The ‘TEST’ phase at CABBI serves as the critical bridge between laboratory‐scale genetic construction and industrial‐scale viability (Figure 4). This stage evaluates how 3P blueprint designs improve TAG concentrations in different plant tissues, impact physiological function, agronomic performance, and how efficiently those lipids can be recovered through downstream processing. Initial testing of these transgenic lines focused predominantly on sugarcane, energycane and sorghum, as established protocols for miscanthus transformation were absent during the earliest DBTL cycles. A consistent finding across these evaluations is that transgenic performance depends strongly on context, with lipid yields varying widely among crop species, tissue types, and environmental growth conditions in ways not fully predicted by controlled laboratory experiments. The results of the test phases so far highlight how different C_4_ crops respond to the metabolic burden of high vegetative TAG accumulation.

**FIGURE 4:**
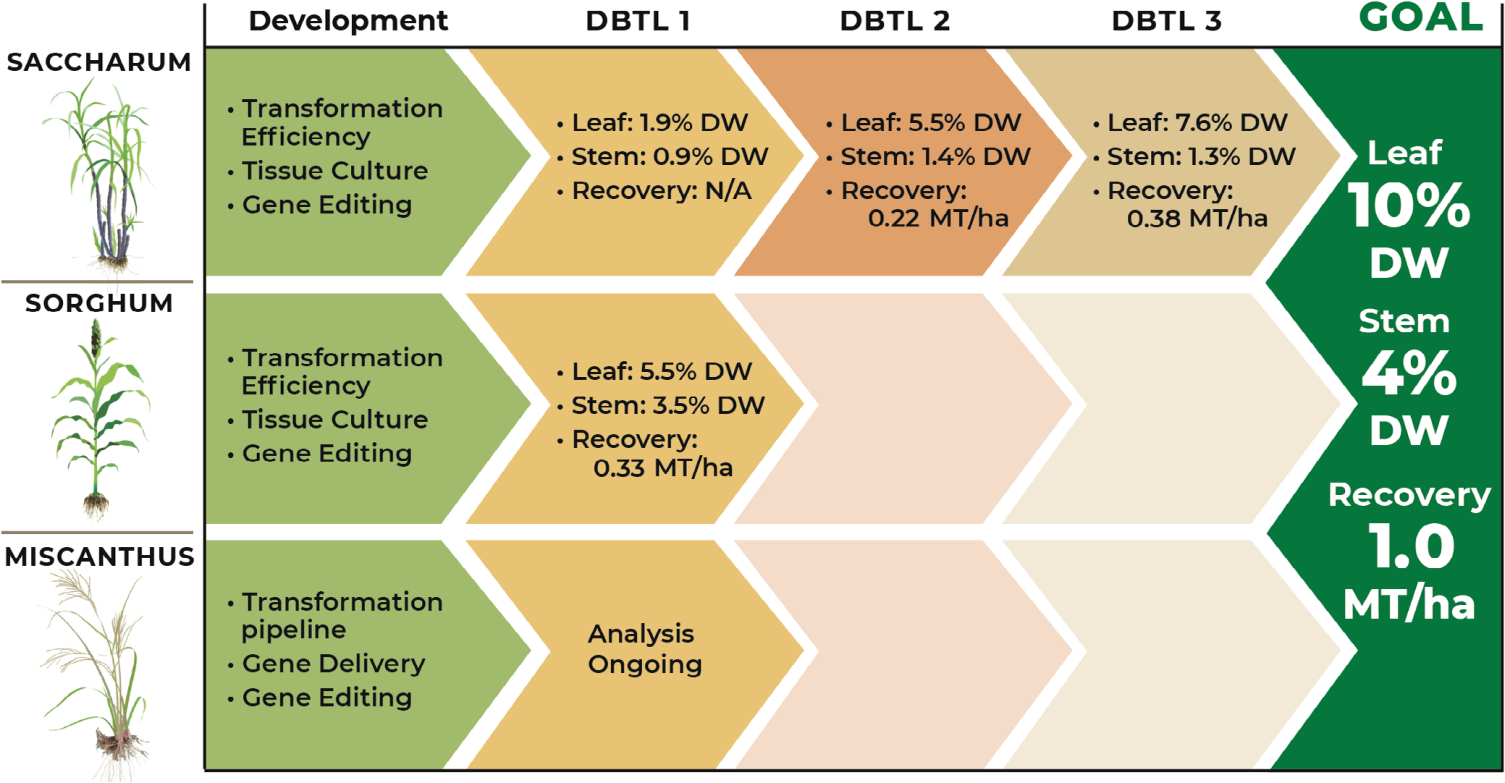
Results and progress from the DBTL cycles for each feedstock crop. Saccharum represents a catch‐all term for sugarcane accessions. The arrows depict progress through DBTL cycles starting with success to develop the feedstock for oil production through to the goal. The goals represent CABBI targets for leaf and stem TAG concentrations and lipid recovery, including a recovery target of 1.0 MT/ha to support commercial viability. Text within each shows the major progression made with either establishing the feedstock chassis or lipid yield from leaves, stem and recovery.

### 4.1 The Saccharum Platform: From Sugarcane to Energycane

The first CABBI crop to complete the design and build phases was oil‐accumulating sugarcane, also called ‘oilcane’. Oilcane plants accumulated TAG up to 1.9% leaf dry weight (DW) and 0.9% stem DW, compared to 0.02% in the stem and leaf of wildtype (Figure 4), using constitutive co‐expression of *WRI1*, *DGAT1‐2* and *OLE1* (Zale et al. 2016). These experiments were subsequently followed by suppressing either SDP1, PXA1, or TGD1 to prevent TAG breakdown and/or reduce the metabolic flux of diacylglycerol into chloroplast lipids. The result was a further increase in TAG accumulation to 5.5% DW in leaves and 1.4% DW in stems (Figure 4) (Parajuli et al. 2020). This TAG accumulation in the stem is particularly important since it translates to a large gain overall due to these tissues being the primary biomass component of CABBI *Saccharum* hybrids. Unfortunately, oilcane plants displayed a biomass penalty of up to 52% observed in many transgenic lines with varying TAG accumulation (Parajuli et al. 2020). Field trials of oilcane showed that transgenes were inherited stably and TAG accumulation was increased by 70‐fold compared to unmodified sugarcane. Notably, lipid yields under field conditions were 2‐fold higher than those under greenhouse conditions (Kannan et al. 2022).

After these initial successes, our engineering focus switched from sugarcane to energycane due to improvements in its transformation technologies. The reason for developing energycane was due to its inherently higher biomass potential, growth range, and resilience in marginal conditions (He et al. 2024; Luo et al. 2022). Genetic designs focused on improving transgene expression by inserting introns into transgenes, which led to 2‐fold higher *DGAT1* transcript levels and a 3.3‐fold increase in TAG accumulation. The intron‐containing *DGAT1* construct was then combined with *WRI1* and *OLE1* to transform energycane, which produced TAG levels of 7.6% DW in leaves and 1.3% DW in stems in replicated field trials (Cao, Kannan, et al. 2023). Energycane plants also showed a reduction in biomass when TAG was accumulated at high levels. However, energycane plants with low levels of TAG accumulation showed no difference in biomass compared to the parentals (Cao, Kannan, et al. 2023). Through the bioprocess evaluation, field‐grown energycane can yield 0.38 MT/ha of lipid, which approaches soybean oil yields of 0.44 MT/ha but remains below CABBI’s 1.0 MT/ha recovery goal. (Figure 4) (Cao, Kannan, et al. 2023; Maitra et al. 2024; Tiseo 2020). Collectively, these achievements represent substantial steps toward engineering energycane with the oil levels and transgene expression stability needed for viable bioenergy production.

### 4.2 The Sorghum Platform: High‐Value Oils and Sustainable Aviation Fuel

While the saccharum platform focused on total lipid volume, sorghum has been designed to produce oils with tailored chemical properties as value‐added products. A primary CABBI target is the production of medium‐chain fatty acids (MCFAs) in grain‐sorghum (TX430) and sweet‐sorghum (Ramada) for sustainable aviation fuel. Initial multigene binary vectors that both stimulate TAG accumulation and promote MCFAs yielded lines with 2.5% dry weight (DW) of TAG in leaves and 2.0% DW of TAG in stems under greenhouse conditions (Park et al. 2025) (Figure 4). However, these lines primarily accumulated common C16 and C18 fatty acid‐containing TAG. This outcome was attributed to insufficient promoter activity driving the gene set and to high MCFA degradation rates in the transgenic tissues. Subsequent field testing of oil‐sorghum in Nebraska during the 2021 and 2022 seasons initially validated the heritability and stability of the high oil trait. Here, the top‐performing events yielded titres of up to 5.5% DW in leaves and 3.5% DW in stems, representing 78‐fold and 58‐fold increases, respectively, relative to wild‐type sorghum (Park et al. 2025). In field conditions, these transgenics produced TAG concentrations in leaves and stems 19–25‐fold higher than in wild type, with yields of up to ~0.45 MT/ha and a multi‐site average of ~0.3 MT/ha (Chen et al. 2026). Our levels were slightly below those previously reported by Vanhercke et al. 2019, but these lines contained more than 25 transgene copies, and the stability of transgenes and phenotypes over multiple generations was not confirmed (Vanhercke et al. 2019).

Following trait validation, CABBI executed a ‘feedstocks‐to‐fuel’ test through expanded field trials in both Illinois and Nebraska over the 2023–2024 growing seasons (Chen et al. 2026). These initial multi‐year evaluations did not detect a biomass yield penalty, leaf gas‐exchange impairment, nutrient dilution, or carbohydrate deficiency associated with the accumulation of vegetative oil in sorghum (Chen et al. 2026). Further, more highly replicated multisite field evaluations of engineered oil sorghum grown across Nebraska and Illinois confirmed that vegetative TAG accumulation can be achieved without impairment of photosynthetic carbon gain, but did detect around an 18% reduction in biomass production in some events (Chen et al. 2026). Notably, biomass penalties were genotype‐dependent, with some grain sorghum lines showing modest reductions, whereas sweet‐sorghum maintained similar biomass levels to wild type (Chen et al. 2026). Together, these results demonstrate that vegetative oil accumulation can be stably expressed across environments, while highlighting the need to optimize the balance between lipid accumulation and biomass productivity.

### 4.3 Bioprocessing of Feedstocks: Lab to Field to Biorefinery

The final validation step in the CABBI pipeline involved the bioprocessing of these engineered feedstocks to recover fuels and co‐products at scale. Deconstructing the lignocellulosic matrix is essential to reduce lignin recalcitrance and improve enzyme access to hydrolyze structural carbohydrates into monomeric sugars for biofuel production. Dilute acid and alkali pretreatments are the most widely tested approaches for developing lignocellulosic biorefineries (Abu‐Ellail et al. 2026). Although dilute acid pretreatment produces pentose sugars, it also promotes the formation of sugar decomposition products and adjusts the pH balance, both of which can inhibit microbial growth and reduce fermentation efficiency (Deshavath et al. 2017). Alkaline pretreatments are effective in delignification and enhance the cellulose digestibility in the subsequent hydrolysis step. However, the loss of hemicellulose along with delignification certainly reduces the overall ethanol yield per ton of biomass (Chandel et al. 2024; Deshavath, Woodruff, and Singh 2024). We found that soaking in aqueous ammonia preserves the hemicellulose and cellulose in the biomass, but saponifies the lipids, thereby reducing lipid recovery from the oil feedstocks (Deshavath et al. 2025). Further, our developed bioprocessing protocol allows for 100% solvent recovery, which aids operating costs when compared with an organosolvent fractionation method that requires energy‐intensive distillation and potential safety risks (Duarte et al. 2024).

Our hydrothermal pretreatment does not require the addition of an external acid or alkali catalyst to deconstruct the lignocellulosic matrix and limits the formation of sugar decomposition products. This leads to higher sugar release, higher ethanol yields during subsequent enzymatic hydrolysis and fermentation, and greater total fatty acid recovery than alkaline and ionic liquid pretreatments (Deshavath et al. 2025). Additionally, hydrothermal pretreatment does not require post‐pretreatment biomass washing, hydrolysate detoxification, or costly nutrients to achieve industrial ethanol titres and yields. In these trials, hydrothermally pretreated energycane, oilcane, sorghum, and miscanthus achieved ethanol yields of 250–290 mL/kg of biomass, using urea as a cost‐effective nitrogen source (Deshavath, Woodruff, Eller, et al. 2024). A significant bioprocessing innovation at CABBI was the integration of a dual‐stack filter into the juicer, enabling substantial wax recovery by separating ‘mud’ from the juice during extraction (Deshavath, Woodruff, and Singh 2024). On a dry‐weight basis, this method produced approximately 53 kg of mud per 1000 L of oilcane juice, yielding up to 25.6% crude wax via hexane extraction (Banerjee et al. 2024). Total juice recovery was increased by 14% through hammer milling of long bagasse strands, the fibrous residue remaining after juice extraction (Deshavath, Woodruff, and Singh 2024). It was further discovered that residual sugars in fibre‐pressed bagasse must be removed prior to hydrothermal pretreatment to prevent the formation of inhibitors such as furfural and 5‐HMF at high temperatures. Using mechanical disc refining increased post‐pretreatment efficiency by increasing the material's surface area and enzyme accessibility. Fed‐batch enzymatic hydrolysis at up to 50% solids yielded sugar concentrations of 200–250 g/L, sufficient to achieve industrial ethanol titres exceeding 40 g/L (Cheng et al. 2019, 2020). Lipid recovery was systematically evaluated following juice fermentation, hydrothermal pretreatment, and enzymatic hydrolysis. Since raw juice contained only 0.01% to 0.1% lipids (Maitra et al. 2022), recovery after fermentation was preferred, leveraging distillation and centrifugation to isolate oil from the stillage. With the hydrothermal pretreatment‐based biorefinery, 55% lipids were recovered from the vegetative tissues of oilcane 1566, corresponding to a lipid yield of 0.22 MT/ha (Maitra et al. 2022). Further oil recovery in oilcane 1565 and transgenic energycane–oilcane line L13 was improved to 83% and 88.7%, yielding 0.35 MT/ha and 0.38 MT/ha of lipids, respectively (Deshavath et al. 2025; Maitra et al. 2024). Overall, the integrated bioprocessing strategy at CABBI demonstrates that high‐biomass C_4_ feedstocks can be successfully deconstructed through hydrothermal pretreatment and tailored extraction to recover high yields of fermentable sugars, lipids for renewable diesel and SAF, and high‐value co‐products like crude wax.

## 5 LEARN: Refining Plant Biodesign and Bioprocessing Through Integrated Feedback

The ‘Learn’ phase at CABBI integrates experimental data, physiological observations, and systems‐level analyses to inform the next generation of biological designs. The value of vertical integration is especially clear in this phase, where combining molecular, physiological, and bioprocessing data across platforms has revealed constraints that would have been difficult to see in isolation. This integrated approach at CABBI has revealed metabolic bottlenecks, physiological trade‐offs, and bioprocessing limits across oilcane, energycane, and oil‐sorghum. By identifying these challenges, we have begun to circumvent metabolic feedback regulation, stabilize biosynthetic enzymes, achieve tissue‐specific expression, and generate targeted catabolic knockouts using cross‐feedstock learning (Table 1).

**TABLE 1:** Summary of LEARN outcomes during the first set of CABBI DBTL cycles.

Learn outcome	Hypothesis	Key factors for future success
Overcome metabolic feedback	3P factors are subject to feedback regulation	Decouple regulatory mechanism
Impact on photosynthetic capacity	Reduced chloroplast lipids	No observed effect
Impairment of specialized cells	Preventing TAG breakdown impairs cellular function	Spatial limitation of 3P factors
Potential cellular toxicity	Lipid intermediates and fatty acid buildup could damage cells	No observed effect
Pleiotropy	Constitutive expression causes off target phenotypes	Spatial limitation of 3P factors
Incomplete bioprocessing protocol	Need to develop feedstock specific pipelines	Hydrothermal treatment
Non‐integrated pipeline	Bioprocessing requires a two‐step process	Semi‐integrated single‐reactor pipeline

### 5.1 Overcoming Metabolic Feedback Regulation

Potential limitations to oil accumulation in vegetative tissues may arise from metabolic imbalances and lipid degradation or turnover processes. WRI1 is considered the master regulator that controls many enzymes that convert sugar into fatty acids (Kuczynski et al. 2022) and is widely used in CABBI transgenics to stimulate TAG production (Chen et al. 2026; Kannan et al. 2022; Parajuli et al. 2020; Park et al. 2025). In oil‐sorghum, transcriptomics showed that many fatty acid biosynthesis genes were highly upregulated as expected from WRI1 overexpression (Park et al. 2025). Further, overexpressing WRI1 in vegatative tissues produced a transcriptional profile akin to that expected for oilseeds (Kuczynski et al. 2022), leading to a divertion of carbon flow from carbohydrates toward fatty acid and TAG synthesis. The result is a disruption of the diurnal cycle of starch that in turn disrupts carbon allocation (Sanjaya et al. 2011; Vanhercke et al. 2014). Recent metabolic data identified a futile cycle of synthesis and breakdown of starch and TAG to occur in tobacco plants accumulating vegetative TAG (Johnson et al. 2025). Similar results were also observed in oil‐sorghum where isotopic tracer experiments showed significantly higher rates of lipid turnover (Park et al. 2025). This discovery was further paired with the identification of a monoacylglycerol lipase gene, which is important in TAG breakdown and was upregulated by more than 60‐fold in transcriptomic analyses (Park et al. 2025). Collectively, these data suggest that metabolic imbalances and futile cycling are likely to severely limit TAG accumulation in vegetative tissues.

How plant cells respond to abnormal lipid‐to‐sugar ratios could also be a reason for low levels of TAG accumulation in vegetative tissues. WRI1 abundance is tightly controlled to prevent TAG production in low trehalose‐6‐phosphate (T6P) conditions. In these conditions, WRI1 is phosphorylated by a SnRK1 kinase, leading to proteasomal degradation (Zhai et al. 2018). Research has shown that WRI1 regulation can be overcome by mutating SnRK1 phosphorylation sites or lysine ubiquitination sites in WRI1 (Zhai et al. 2017). CABBI researchers discovered that of these two mutations, mutating SnRK1 target sites can disrupt the DNA‐binding site and inactivate the protein while mutation of ubiquitin conjugation sites stabilized WRI1 and increased TAG accumulation (Zhai et al. 2017, 2021). Similar types of regulatory domains have also been discovered within the genes that act as ‘PULL’ and ‘PROTECT’ factors in CABBI oil‐accumulating crops. The DGAT enzyme also shows increased activity upon mutation of the SnRK1 phosphorylation site (Xu et al. 2008). Mutation of a ubiquitin‐conjugation site in an oleosin from *Sesamum indicum* stabilized the proteins and resulted in increased levels of leaf oil (Anaokar et al. 2024). The research into WRI1, which promotes lipid transfer from the ER to the chloroplast and thereby depletes the diacylglycerol pool available for TAG synthesis., DGAT, and OLE protein structure demonstrates that we can alter proteins or use distantly related variants to mitigate the effect of regulatory pathways and optimize TAG accumulation in future DBTL cycles.

### 5.2 Identifying the Biological Causes of Yield Drag

Crops engineered for vegetative TAG must maximize yield potential for economic viability. Initial proof‐of‐concept studies in tobacco suggested that leaf tissue could accumulate 14% TAG without impacting biomass (Vanhercke et al. 2014). However, transferring these technologies into C_4_ grasses has revealed instances of up to 52% biomass reduction in oilcane or energycane (Cao, Kannan, et al. 2023; Kannan et al. 2022; Parajuli et al. 2020; Zale et al. 2016) and up to 18% in grain sorghum (Chen et al. 2026). However, the oil‐accumulating line of sweet sorghum showed similar levels of biomass compared to wildtype (Chen et al. 2026; Park et al. 2025). An initial hypothesis is that oilcane and energycane showed reduced biomass due to accumulating higher TAG than oil‐sorghum (Parajuli et al. 2020; Park et al. 2025). This is also supported by energycane transgenics with lower TAG accumulation displaying equal biomass yields under field conditions (Cao, Kannan, et al. 2023). However, this is unlikely the sole factor since oilcane lines with higher TAG levels than those in energycane but lower than the highest observed levels still showed reductions in biomass (Parajuli et al. 2020).

One hypothesis is that increasing TAG production reduces chloroplast lipid synthesis, thereby reducing photosynthesis. This occurs because many chloroplast lipids are derived from ER‐derived precursors shared with TAG assembly (Ohlrogge and Browse 1995). Therefore, shifting the metabolic flux more in favour of TAG production could reduce photosynthetic capacity by lowering chloroplast lipid levels. Indeed, reduced chloroplast lipid production was shown by metabolic flux analysis in oil‐accumulating tobacco plants with reduced biomass (Vanhercke et al. 2017; Zhou et al. 2020). However, oil‐sorghum displayed no reduction in biomass and had similar levels of leaf chloroplast lipids compared to wildtype (Park et al. 2025). Alternatively, shifts in the bound fatty acid species of chloroplast lipids represent another mechanism that could impact plant biomass. Oilcane, oil‐sorghum, and oil‐producing tobacco all show reductions in 18:3 fatty acids compared to parental lines (Parajuli et al. 2020; Park et al. 2025; Zhang et al. 2025). This is important because fatty acid composition of chloroplast lipids is important in stabilizing the photosystems within the thylakoid membrane and could lead to lower photosynthetic capacity (Hölzl and Dörmann 2019). These data are consistent with TAG accumulation in leaves coming at the expense of chloroplast lipids in at least some feedstocks, which may limit biomass production.

Another possibility is that preventing TAG breakdown impacts the cellular function of specialized cell types. One example of these specialized cell types is guard and subsidiary cells in the monocot stomatal complex. These cells play a crucial role in adjusting the stomatal pore to control plant CO_2_ and water loss for optimal water use efficiency (Crawford et al. 2025). In Arabidopsis, TAG has been implicated in providing ATP for stomatal movement in response to light and when under abiotic stress (Korte et al. 2023; McLachlan et al. 2016). One study has shown that excessive leaf oil and overly large lipid droplets reduced stomatal conductance (Murphy et al. 2025), which is highly correlated with the size of the stomatal aperture (Crawford et al. 2025). Given the role stomata play in leaf physiology, it is entirely possible that limiting stomatal aperture by preventing TAG breakdown could decrease photosynthetic carbon gain and thereby reduce biomass. A recent report commented that large lipid droplets were observed within the stomata of oil‐accumulating energycane (Cao, Kannan, et al. 2023). However, to date, no study has examined the role of TAG in the stomata of C_4_ grasses, and the exact impact of decreasing TAG breakdown remains unknown.

Lastly, increased flux through lipid biosynthetic pathways can lead to the accumulation of intermediates within metabolic pathways and/or free radicals that exert toxic effects on the cell. Indeed, expressing WRI1 in the vegetative tissue of *Brachypodium distachyon* led to increased levels of free fatty acids, resulting in increased cellular toxicity (Yang et al. 2015). One explanation for these results is that increased fatty acid and lipid biosynthesis likely disturbs the balance between energy cofactor production (ATP and NADPH) by photosynthetic light reactions (Clark and Schwender 2022). The build‐up of free fatty acids has been shown to affect vegetative tissue growth in various mutants that alter lipid metabolism by altering flux from source to sink (Fan et al. 2013, 2026). If enhanced lipid synthesis is paired with increased lipid degradation, increased fatty acids and elevated β‐oxidation activity may lead to the excessive production of cytotoxic reactive oxygen species. While reactive oxygen species have considerable roles in plants, they are still highly damaging when uncoupled from tight regulatory networks (Mittler et al. 2022). Several reports have already shown how disrupting fatty acid β‐oxidation leads to cellular damage through uncontrolled reactive oxygen species (Yu et al. 2019). Each of these hypotheses still requires further investigation to fully inform future designs. However, the integrated nature of CABBI enabled the early identification of these constraints due to each DBTL cycle representing a full lab‐to‐field pipeline.

### 5.3 Refining Bioprocessing Efficiency

Bioprocessing feedback loops have also provided essential ‘LEARN’ signals for feedstock design. One major observation was that residual sugars in fibre‐pressed bagasse are converted into fermentation inhibitors such as furfural and 5‐HMF during high‐temperature hydrothermal pretreatment. While conventional approaches require removal of these sugars through washing, CABBI researchers demonstrated that hydrothermal pretreatment can be performed without washing to generate and recover these platform chemicals, which are then recovered via nanofiltration (Jia et al. 2024). Furthermore, a newly developed semi‐integrated bioprocess, combining 18 h of enzymatic hydrolysis with fermentation in a single reactor, achieved complete utilization of glucose and xylose at high solid loadings of 25%–30% (Deshavath, Woodruff, Eller, et al. 2024). This approach produced ethanol yields of 250–290 L/tonne of biomass while dramatically reducing the required inputs of NaOH and urea. These advancements are being fed back into the ‘DESIGN’ phase to create ‘processing‐friendly’ feedstocks where cell wall composition is modified alongside lipid content to enhance enzymatic accessibility and oil release. Collectively, the physiological, system‐level, and bioprocessing insights gained across these platforms are now informing design for additional DBTL cycles.

## 6 Emerging Technologies: Accelerating Design–Build–Test–Learn Cycles

Technological advances at CABBI have established pipelines and developed protocols to streamline engineering feedstock crops. However, engineering TAG‐accumulating field‐grown crops remains a labour‐intensive endeavour. Each phase of the DBTL cycle introduces delays that constrain overall efficiency because it requires generating transgenic lines for preliminary evaluations and final field trials. Therefore, to achieve a sustainable bioeconomy, CABBI has begun integrating computational tools and advanced engineering to shorten the transition from laboratory discovery to field‐proven feedstock (Figure 5).

**FIGURE 5:**
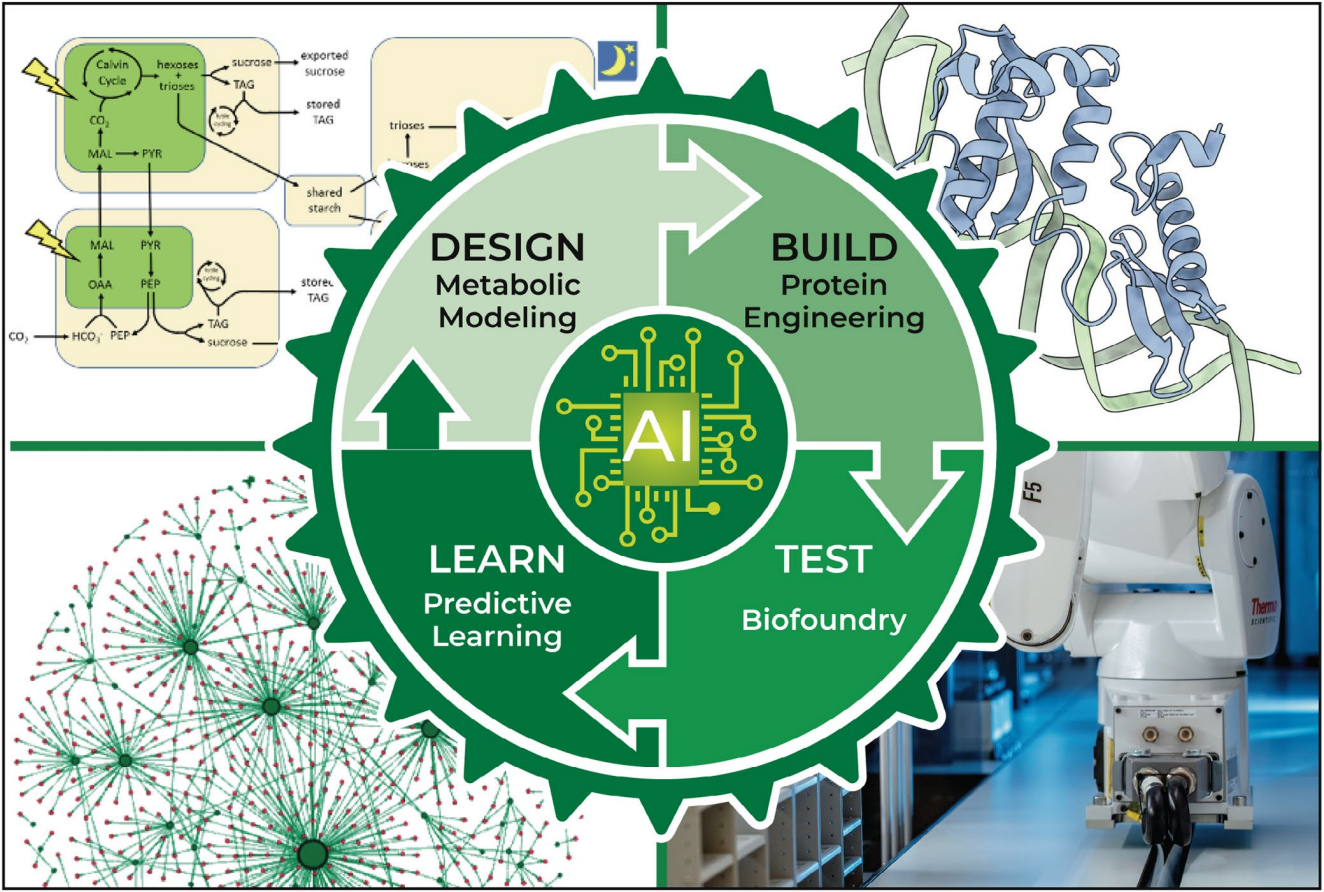
Integrating emerging technologies to enhance DBTL turnover. Graphic showing how emerging technologies can be integrated into the Design (Metabolic Modelling), Build (Protein Engineering), Test (Biofoundry), Learn (Predictive Learning) cycle. The centre represents how all these can be supplemented with the predictive power of AI.

### 6.1 Predictive Modelling and Biofoundry Screening

A major challenge facing the current DBTL cycle is the lack of a reliable modelling framework. A genetic construct designed to enhance TAG biosynthesis may have complex, context‐dependent effects at the whole‐plant level. Gene expression levels and metabolic behaviour can vary across cell types and developmental stages. Although frameworks for model‐assisted metabolic engineering in plant cell cultures have been developed (Correa et al. 2020; Schwender et al. 2014; Schwender and Hay 2012), these models are not yet sufficiently robust or predictive at the whole‐plant scale. Overcoming this limitation could be achieved by creating a test bed to produce metabolomics, transcriptomics, and fluxomics datasets as training sets to feed into model‐assisted frameworks designed to predict construct design to increase TAG accumulation.

Biofoundries, automated platforms for high‐throughput screening that feed data into predictive modelling, represent a powerful new avenue for accelerating metabolic flux models and DBTL cycles. CABBI researchers currently benefit from two biofoundries at the University of Illinois, which enable rapid selection of promising construct designs for plant protoplasts (Dong et al. 2025). These biofoundries are coupled with FAST‐PB (Fast, Automated, Scalable, and high‐throughput Pipeline for plant Bioengineering), an automated plant bioengineering system that integrates robotics, computer‐aided design, and informatics. CABBI has used the FAST‐PB platform to enable high‐throughput identification and validation of the effects of WRI1, DGAT1, and oleosin overexpressio*n* on lipid metabolism in *Nicotiana benthamiana* cells (Dong et al. 2025). A specific biofoundry uses the iBioFAB (Illinois Biological Foundry for Advanced Biomanufacturing) robotic laboratory to automate Golden Gate cloning, PCR preparation, and *E. coli* transformation, enabling the parallel construction of 96 vectors. The iBioFAB is designed specifically to integrate with AI tools such as the protein‐engineering workflow ESM2. Using this tool, CABBI researchers completed four rounds of variant screening within 4 weeks, evaluating approximately 1000 variants across two proteins (Singh et al. 2025). The development of these shows that vertical integration within CABBI can stimulate innovation in adjacent fields.

### 6.2 Increasing Transformation Efficiency

Increasing the efficiency of tissue culture and transformation is a central goal for increasing success when translating discoveries from model species to crops. The development of ternary vector systems incorporating additional virulence genes is particularly promising, as these systems have increased transformation efficiencies by between 29% and 164% in sorghum (Jeong et al. 2025). This technology can be paired with engineered thymidine auxotrophic *Agrobacterium* strains to reduce post‐cocultivation antibiotic loads and newly developed biolistic flow‐guiding barrels that improve gene transfer by more than 10‐fold (Thorpe et al. 2025) and can increase the throughput of DBTL cycles.

In gene modification, base editors have enabled precise conversions without double‐strand breaks in soybean (Cai et al. 2020), rice (Ren et al. 2021), and sorghum (Zhou et al. 2026). Prime editing technologies also show promise for polyploid crops, with successful applications in tetraploid potato (Perroud et al. 2022) and hexaploid wheat (Ni et al. 2023). DNA‐free editing using ribonucleoprotein complexes provides a potential regulatory advantage in some global frameworks because edited plants can be produced without transgene integration (Svitashev et al. 2016). CRISPR‐derived diversification systems have also enabled synthetic directed evolution approaches for engineering plant proteins and traits in Arabidopsis and rice (Butt et al. 2022; Dong et al. 2024). This raises the possibility of engineering TAG biosynthetic enzymes in vivo with enhanced catalytic properties or altered regulatory behaviour in polyploid species. Currently, these technologies have mostly been used to improve the chassis of target crop species, with one notable example being the mutation of *LIGULELESS‐1* in sorghum and sugarcane to improve yield by altering leaf angle (Brant et al. 2021, 2024). Collectively, these emerging technologies can accelerate DBTL cycles and future oil yields by increasing transformation success, chassis design, and TAG accumulation.

### 6.3 Advanced Regulatory Control Systems

The observed biomass reduction in oilcane lines indicates that oil accumulation can adversely affect molecular and physiological processes. A goal for CABBI is now to explore how to limit off‐target effects from constitutively expressed transgenes, as widely detailed in the literature (Kummari et al. 2020). One way to mitigate these effects in gene function studies is to use inducible promoters. CABBI has employed inducible promoters that are active only under specific conditions such as elevated temperature (Qiande and Altpeter 2025). However, these promoters may not always activate under field conditions, leading CABBI to explore ways to restrict transgene expression using tissue‐specific promoters. Fortunately, CABBI has shown that promoters that drive expression in early leaf development, but not in mature leaves or stems, avoid unwanted effects on leaf and stem physiology (Lunn et al. 2026). The challenge is identifying conserved tissue‐specific promoters in grasses. To address this challenge, CABBI developed unique single‐cell transcriptomic pipelines to map specific cell types in sorghum (Fu et al. 2024). These datasets enabled the identification of stem‐specific genes, whose promoters can be adapted to achieve targeted transgene expression patterns in sorghum (Fu et al. 2025). CABBI has successfully identified candidates through a combination of RNA‐seq atlases, genomic and phylogenetic data, and quantitative PCR analysis (Wang et al. 2021). However, screening libraries generated from these datasets remains challenging, as testing requires generating transgenic plants with fluorescent markers for cell‐type identification and gene‐expression constructs.

Synthetic promoter technologies offer an attractive solution for limiting transgene expression spatially because they do not require identifying truly tissue‐specific promoters. In this approach, multiple cis‐regulatory elements (CREs) work together to control gene expression levels and specificity. Altering the number, orientation, and spacing of CRE modules can modulate expression strength and tissue specificity (Nayak et al. 2025). A key advantage of CRE‐based promoters is that they come from various organisms, have minimal repeat sequences, and low homology to each other, which minimizes the risk of transgene silencing in multi‐gene constructs (Nayak et al. 2025). Researchers have also combined CRE modules with synthetic transcription factors to build synthetic gene circuits that operate through Boolean logic (Brophy et al. 2022). Further, the advent of machine learning has enabled the functional prediction of CRE function using large genomic datasets (Li et al. 2024). Coupled with the emerging field of single‐cell and spatial omics studies, these tools could create truly orthogonal, multiplexed regulation, enabling precise, scalable modification of multi‐gene expression profiles in developmentally regulated or inducible contexts.

## 7 Conclusion: The Future of the C_4_ Bioeconomy

The successful hyper‐accumulation of TAG in vegetative tissues of C_4_ grasses represents an important advance toward scalable production of renewable diesel and sustainable aviation fuel. By developing the feedstocks‐to‐fuel pipeline, CABBI has demonstrated that feedstocks such as energycane and oil‐sorghum can achieve lipid productivity close to conventional soybean yields. Importantly, this progress did not come from a single breakthrough, but from coordinated solutions to constraints in metabolic regulation, crop performance, and downstream deconstruction. A defining outcome of this effort is that centre‐scale DBTL cycles reveal discoveries that are difficult to achieve in isolated research programs. Across multiple feedstocks, DBTL iterations revealed that (i) vegetative tissues actively counter‐regulate lipid accumulation through enhanced TAG catabolism, (ii) yield drag can arise from spatial mis‐expression, photosynthetic disruption, or membrane‐associated trade‐offs rather than simple carbon limitation, and (iii) lipid recoverability and inhibitor formation impose constraints that must be addressed during biological design rather than retrofitted during processing.

Looking forward, TEST and LEARN insights from early‐generation high‐TAG platforms are reshaping subsequent DBTL cycles in ways that would not have been possible without the cross‐scale data integration enabled by centre‐scale research. Systems‐level analyses are aimed at stabilizing key biosynthetic proteins, overcoming feedback regulation, suppressing lipid breakdown, and restricting transgene expression to cell types or developmental stages where oil accumulation is least likely to impair biomass yield. In parallel, emerging tools such as precision genome editing, biofoundry‐enabled construct screening, and machine‐learning‐assisted regulatory design are expected to accelerate iteration and improve performance predictability across growth environments. More broadly, the challenges encountered here, spanning metabolic regulation, crop transformation, field agronomy, and downstream processing, illustrate why complex traits in non‐model crops are difficult to advance through siloed research efforts. Together, these advances support high‐biomass C_4_ crops as a foundation for a resilient bioeconomy, while showing that continued progress will depend on DBTL frameworks that link biological design with field performance and process compatibility.

## Author Contributions

D.L., E.B.C., F.A., J.Sh., and V.S. conceived and designed the outline of the manuscript. D.L., K.P., N.N.D., J.D., K.S., J.Sc., J.G., A.D.B.L., N.M.B., V.S., F.A., J.Sh., and E.B.C. contributed to the writing of sections relating to their field of expertise. All authors were involved in writing, reading, and approving the final manuscript.

## Funding

This work was funded by the DOE Center for Advanced Bioenergy and Bioproducts Innovation (US Department of Energy, Office of Science, Biological and Environmental Research Program under Award Number DE‐SC0018420). Any opinions, findings, and conclusions or recommendations are those of the author(s) and do not necessarily reflect the views of the US Department of Energy.

## Disclosure

Gemini AI was used to guide word‐count reduction in an early draft. All other drafts were produced without AI assistance, and no AI was used in constructing the figures.

## Conflicts of Interest

John Shanklin has a financial interest in AtTag Bio Inc.

## Data Availability

Data sharing not applicable to this article as no datasets were generated or analysed during the current study.
